# Challenging diagnosis of a coronary arteriovenous malformation: a case report

**DOI:** 10.1186/s44215-025-00233-2

**Published:** 2025-11-14

**Authors:** Yuto Imaizumi, Hiroo Uehara, Manato Saitoh, Naomi Ozawa, Makoto Ono, Masateru Uchiyama, Tomohiro Imazuru, Tomoki Shimokawa

**Affiliations:** 1 Department of Cardiovascular Surgery, Teikyo University, 2-21-1 Kaga, Itabashi-Ku, Tokyo 173-8606 Japan; 2 Department of Cardiovascular Surgery, Sakakibara Heart Institute, 3-16-1 Asahicyo, Fuchushi, Tokyo 183-0003 Japan

**Keywords:** Coronary arteriovenous malformations, Coronary arteriovenous fistula, The International Society for the Study of Vascular Anomalies (ISSVA) classification

## Abstract

**Background:**

Accurately distinguishing between coronary arteriovenous malformations (CAVMs) and fistulas is challenging. We encountered a case of preoperatively undiagnosed CAVM.

**Case presentation:**

We report the case of a 45-year-old woman diagnosed with cardiac enlargement during a routine health examination. Transthoracic echocardiography showed normal left ventricular systolic function with an ejection fraction of 68%, normal valve function, and a well-defined mass adjacent to the posterior and inferior cardiac walls of the left ventricle. Transesophageal echocardiography revealed a solid mass with a partial honeycomb structure adjacent to the posterior cardiac wall of the left ventricle. A contrast-enhanced computed tomography (CT) scan revealed a solid mass (115 × 79 × 30 mm) with heterogeneous enhancement adhering to the posterior wall and arterial inflow into the mass. Angiography of the right coronary artery revealed mild contrast agent pooling in the venous phase, suggesting the presence of a nutrient vessel in the mass. Cytological examination and histopathological diagnosis after CT-guided needle biopsy revealed Class III tumor with no malignant findings. Considering the challenges in diagnosis with the current tests and the potential risk of cardiac tamponade caused by repeated biopsies, we adopted a policy that tumorectomy should be performed to elucidate the diagnosis. Additional surgical procedures could then be conducted if the intraoperative diagnosis showed a malignant tumor. Intraoperative findings showed that the mass was firmly adherent to the heart between the inferior and lateral walls of the left ventricle. The intraoperative diagnosis of the tumor showed no malignancy. Planned tumorectomy was performed after careful dissection of tumor adhesion. Part of the left marginal vein of the coronary sinus that could not be dissected from the adhesions was resected. Immunohistochemical studies demonstrated CD31+ vascular endothelium, suggesting that the mass was an arteriovenous malformation. The patient’s postoperative course was uneventful, without any signs of recurrence.

**Conclusion:**

This case highlights the difficulty of diagnosing CAVM preoperatively and shows that when tumor imaging is ambiguous, CAVM should be considered and early surgical exploration is crucial.

## Background

The incidence of cardiac tumors has been reported to be 0.0017–0.33% based on autopsy studies [1]. Coronary arteriovenous malformations (CAVMs) are extremely rare according to the International Society of Vascular Anomalies (ISSVA) classification [2]. Furthermore, the accurate preoperative diagnosis of coronary artery anomalies is challenging. Herein, we present a rare case of CAVM that proved difficult to diagnose before surgery, highlighting the limitations of current imaging modalities and the diagnostic and therapeutic, value of surgical resection.

## Case presentation

**Fig. 1:**
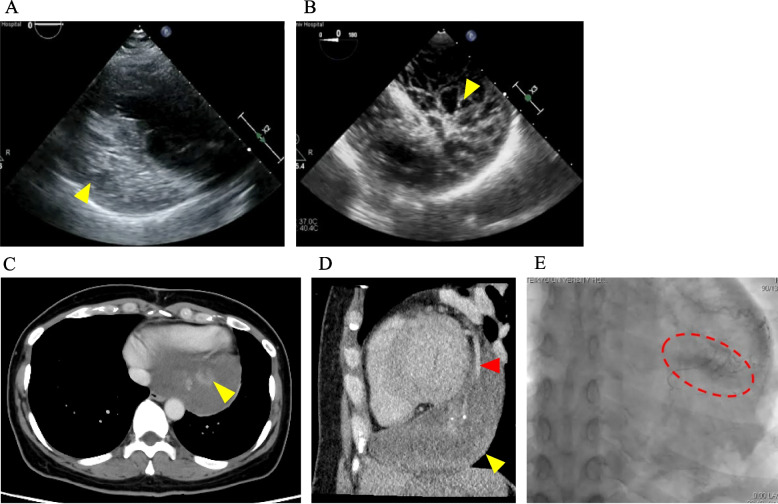
Preoperative findings. **A** Transthoracic echocardiography. **B** Transesophageal echocardiography. **C**, **D** Computed tomography. A tumor is shown as a yellow triangle and arterial inflow is shown as a red triangle. **E** Coronary angiography. Pooling of contrast agent is shown as a red circle

The patient was a 45-year-old woman with no medical or family history. She was diagnosed with cardiac enlargement during a routine health examination and transferred to our institution for further investigation of an undiagnosed cardiac mass. She had average intelligence, and her height, weight, and body surface area were 166.5 cm, 59.8 kg, and 1.667 m^2^, respectively. Laboratory values on admission showed mild coagulation abnormalities (D-dimer, 4.5 µg/mL; fibrin/fibrinogen degradation products, 9.9 µg/mL) and an elevated cardiac marker (N-terminal pro-brain natriuretic peptide, 200.0 pg/mL). IL-2 receptor was 177 U/mL.　However, inflammatory and tumor markers were within the normal range. Transthoracic echocardiography (TTE) showed normal left ventricular systolic function with an ejection fraction of 68%, normal valve function, and a well-defined mass adjacent to the posterior and inferior cardiac walls of the left ventricle (Fig. 1A). Transesophageal echocardiography (TEE) revealed a solid mass with a partial honeycomb structure adjacent to the posterior cardiac walls of the left ventricle (Fig. 1B). Initial contrast-enhanced computed tomography (CT) scan revealed a solid mass (115 × 79 × 30 mm) with heterogeneous enhancement adherent to the posterior wall (Fig. 1C) and arterial inflow into the mass (Fig. 1D). Electrocardiography (ECG) and positron emission tomography-CT were unremarkable. Cardiac multi-detector row CT (MDCT) revealed no abnormal findings in the coronary arteries. However, angiography of the right coronary artery revealed mild contrast agent pooling in the venous phase, suggesting the presence of a nutrient vessel for the mass in the distal part of the right coronary artery (Fig. 1E). Magnetic resonance imaging (MRI) revealed a smooth-surfaced mass located extramyocardially along the posterior wall of the left ventricle and the inferior wall of the right ventricle. On T1-weighted images, the mass exhibited predominantly low signal intensity with focal areas of high signal intensity suggestive of intralesional hematoma. T2-weighted images demonstrated multiple internal septa with high signal intensity. Fluorodeoxyglucose-Positron Emission Tomography/CT (FDG-PET/CT) revealed a soft tissue dense tumor that was widely adjacent to the left border of the heart, and was suspected to be of pericardial origin due to its localization. Furthermore, the SUVmax was 2.0, and no strong malignant findings were suspected. Cytological examination and histopathological diagnosis after CT-guided needle biopsy revealed Class III tumors and no malignant findings. Because blood tests did not reveal any malignant findings, and based on TTE, TEE, CT imaging, MRI, and FDG-PET/CT hemangioma, pericardial cyst with hemorrhage, teratoma, ganglioneuroma, and coronary artery anomalies were initially suspected. Due to the limitations of current diagnostic modalities and the fact that intracardiac masses, including those within the pericardial space, may not present with typical symptoms such as cardiac tamponade if they enlarge slowly over time, clinical symptoms alone may not be sufficient for accurate diagnosis. Moreover, cardiac tumors often lack characteristic imaging findings, making definitive diagnosis challenging. Therefore, we opted to perform surgical resection of the tumor to establish a definitive diagnosis.

**Fig. 2:**
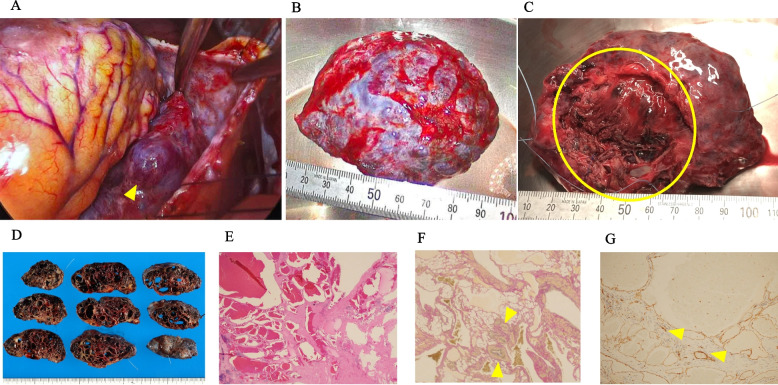
Intraoperative findings and pathological assessment. **A** After incision of the pericardium. **B** A tumor (97 × 70 × 30 mm) was resected. **C** Heart attachment surface. Only the area of the tumor indicated by the circle was attached to the heart. **D** Pathological specimens. **E** Histologic examination (hematoxylin–eosin staining) of the tumor. **F** Immunohistochemical examination with Elastica van Gieson staining. An Elastica van Gieson staining vascular endothelium is shown as yellow triangles. **G** Immunohistochemical examination with anti-CD31 monoclonal antibody. A CD31 + vascular endothelium is shown as yellow triangles

The surgical strategy for this case was to perform only pathological specimen collection if the intraoperative diagnosis showed clear malignant findings through thoracotomy. In addition, if there was no adhesion and it seemed possible to remove the tumor, the incision would be extended and surgery would be performed. In addition, 5Fr. sheaths were inserted into the left common femoral artery and vein before surgery to prevent bleeding. An anterolateral small thoracotomy of 8 cm through the left 5th intercostal space was performed under general anesthesia. The procedure was performed in the 60° right lateral decubitus position, which allowed adequate exposure of the tumor after opening the pericardium (Fig. 2A). The tumor was firmly adherent to the left ventricular wall, but no gross evidence of myocardial involvement was observed. However, the tumor was firmly adherent to the inferior and lateral walls of the left ventricle through fibrovascular tissue. The non-adherent tumor was partially resected and submitted for intraoperative pathological examination. The intraoperative diagnosis of the tumor showed no malignancy. It was determined that an 8-cm incision would be sufficient for resection; therefore, the incision was not extended and the procedure was continued. Hemostasis of the bleeding areas was achieved using 4–0 Prolene sutures. A planned tumorectomy was performed after careful dissection of the tumor adhesions (Fig. 2B and C). The distal side of the posterior descending coronary artery and part of the left marginal vein of the coronary sinus that could not be dissected from the adhesions was resected. The operation time was 246 min. The patient’s postoperative course was uneventful, without any serious perioperative complications. Pathological examination of the resected tumor revealed a multilocular cyst filled with hemorrhagic fluid (Fig. 2D). Hematoxylin–eosin and Elastica van Gieson staining of the tumor showed cystic spaces filled with red blood cells and intimal hyperplasia, respectively (Figs. 2E and F). Immunohistochemical studies demonstrated a CD31^+^ vascular endothelium (Fig. 2G). The proliferating vascular walls exhibited varying thicknesses and consisted of a mixture of muscular vessels with well-defined smooth muscle layers and thin-walled vessels. Intervening between the vascular channels were irregular bundles of smooth muscle and adipose tissue. This complex admixture of CD31 + endothelium and vessels of various sizes suggests continuity between these vessels and is consistent with an arteriovenous malformation. No nidus was identified in the specimen. In the present case, the findings suggest that the mass represents an arteriovenous malformation, categorized as a simple vascular malformation under the ISSVA classification. Angiomyolipoma and solitary fibrous tumor were considered in the differential diagnosis. However, angiomyolipoma was deemed unlikely, as immunohistochemical staining for HMB45 and Melan-A was negative. Solitary fibrous tumor was also considered unlikely due to the absence of STAT6 and CD34 expression. Postoperative TTE, TEE, and contrast-enhanced CT revealed no clear remnant tumor on the cardiac wall, and the patient was discharged on postoperative day 9. No tumor recurrence was observed during the 2.5-years postoperative follow-up.

## Discussion

Arteriovenous malformations are classified as simple vascular malformations by the ISSVA [2]. In particular, CAVM is extremely rare, with an incidence of 0.002% and accounts for approximately half of all coronary artery anomalies. Although 55–80% of CAVMs occur as isolated cases, their comorbidities include congenital heart diseases such as atrial septal defect, tetralogy of Fallot, and patent ductus arteriosus [3]. Invasive coronary angiography (CAG) has been the standard modality for diagnosing coronary artery anomalies, including CAVMs, but it has limitations in assessing the three-dimensional anatomy. The advent of high-resolution cardiac-gated MDCT has improved the noninvasive detection of CAVMs, even in asymptomatic patients. According to MDCT findings, CAVMs most commonly originate from the right coronary artery (55%), followed by the left anterior descending artery (35%). Approximately two-thirds drain into the right atrium or ventricle, while drainage into the left heart chambers is rare (3–5%) [3]. One of the preoperative differential diagnoses in patients with CAVM is coronary arteriovenous fistula (CAVF). The typical imaging findings of CT and MRI are generally reported to include the presence of a nidus (an enlarged feeding artery and draining vein). However, the ISSVA classification does not clearly distinguish them on imaging [4, 5]. Differential diagnosis of arteriovenous malformations (AVMs) are challenging to diagnose definitively because the specific components of AVMs, such as arterial vessels, draining veins, and nidus, can vary significantly between individual case [6, 7]. As reported on diagnostic imaging using MDCT, the predilection site of CAVMs was the right coronary artery (55%), followed by the left anterior descending artery (35%). Fistulas draining into the right atrium or ventricle accounted for two-thirds of the total, whereas fistula draining into the left atrium or ventricle accounted for only 3–5% [3]. In this case, preoperative TTE and TEE revealed a solid cardiac tumor, partially exhibiting a honeycomb-like structure. Preoperative MDCT revealed a single inflow vessel supplying the solid tumor attached to the posterior wall of the left ventricle. Preoperative CAG showed pooling of contrast medium in the venous phase; however, no definitive outflow veins were identified. Based on these preoperative findings, no significant abnormalities were observed in the coronary arteries, and CAVMs were not suspected. Unexpectedly, intraoperative findings revealed that the distal posterior descending coronary artery and the left marginal vein of the coronary sinus had true anatomical continuity between the tumor and cardiac tissue, which was the primary site of the CAVMs. This case highlights the limitations of preoperative imaging in diagnosing cardiac tumors and underscores the diagnostic and therapeutic value of surgical intervention.

Currently, there is no consensus on the management of CAVM and surgical intervention. To date, symptomatic patients have undergone surgical intervention. Asymptomatic patients with i) a shunt fraction of 30% or more, ii) evidence of ischemic changes on ECG, iii) the development of pulmonary hypertension or heart failure, iv) a history of bacterial endocarditis, or v) a risk of aneurysm (fistula aneurysm) rupture tend to undergo surgery [8]. Depending on their localization, AVMs have been treated with transcatheter embolization, especially in cases associated with stable hemodynamics, or surgical resection in challenging cases with a large tumor [9–11]. Importantly, asymptomatic patients with stable hemodynamics and none of the aforementioned findings have been managed conservatively, without surgical intervention [12]. In contrast, since there has been an intriguing report of sudden death in an asymptomatic patient with CAVM [13], careful consideration is required regardless of the presence or absence of symptoms. In our case, we performed a planned tumorectomy based on the aforementioned studies and preoperative diagnosis because the asymptomatic patient had an undiagnosed large mass. Although CAVMs are often diagnosed using MDCT, this case highlights the need to recognize that some CAVMs may not be visualized on MDCT. It also emphasizes the importance of considering CAVMs in the differential diagnosis of cardiac tumors. This case underscores the limitations of preoperative imaging in the evaluation of cardiac tumors and the diagnostic and therapeutic value of surgical intervention.

## Conclusion

This case provided valuable insights into the preoperative diagnostic difficulties of CAVM, highlighting the importance and challenges of accurate imaging diagnosis.

## Data Availability

The datasets used are available from the corresponding author on reasonable request.

## Abbreviations

AVMs: arteriovenous malformations
CAG: coronary angiography
CAVMs: coronary arteriovenous malformations
CAVFs: coronary arteriovenous fistulas
CT: computed tomography
ECG: electrocardiogram
FDE-PET/CT: Fluorodeoxyglucose-Positron Emission Tomography/CT
ISSVA: International Society for the Study of Vascular Anomalies
MDCT: multi-detector row CT
MRI: Magnetic resonance imaging
TEE: transesophageal echocardiography
TTE: Transthoracic echocardiography
